# Molecular basis for chikungunya virus recognition of a mosquito-specific receptor

**DOI:** 10.64898/2026.07.28.741352

**Published:** 2026-07-29

**Authors:** Xiaoyi Fan, Wanyu Li, Jesse S. Plung, Jessica A. Plante, Ellie M. Hajovsky, Nisha Tapryal, Jennifer Diaz, Nicholas C. Hazell, Chenggong Ji, Zhiqi Liu, Catherine E. Hammond, Scott C. Weaver, Kenneth S. Plante, Jonathan Abraham

## Abstract

Alphaviruses are arthropod-borne viruses that recognize cellular receptors in both vertebrate hosts and mosquito vectors to complete their transmission cycle, yet how they maintain recognition of receptors across evolutionarily divergent host species remains unresolved. Among alphaviruses, chikungunya virus (CHIKV), which is primarily vectored in urban settings by *Aedes* species mosquitoes, is the most widespread, and causes explosive outbreaks that can involve hundreds of thousands to millions of cases annually. The cell adhesion protein Lachesin is a mosquito-specific cellular receptor for CHIKV and multiple other arthritogenic alphaviruses. The envelope E2–E1 glycoproteins of these alphaviruses broadly recognize Lachesin orthologs from diverse mosquito species, but not other insects or arachnids. Lachesin genetic manipulation to prevent mosquito virus infection without interfering with endogenous receptor function could have a major impact for CHIKV control. Here, we determined high-resolution cryo-electron microscopy (cryo-EM) structures of alphaviruses bound to *Aedes albopictus* Lachesin. Comparative analysis of Lachesin-bound CHIKV, Semliki Forest virus (SFV), and Middelburg virus (MIDV) revealed that these three genetically divergent viruses use a similar surface to recognize Lachesin domain 1, but with reorganized E2–E1 glycoprotein contact residues. We show that a soluble *Ae. albopictus* Lachesin receptor decoy protein blocks the E2–E1-mediated entry of CHIKV and other arthritogenic alphaviruses into mammalian cells with greater breadth than a vertebrate receptor MXRA8 decoy and protects against lethal SFV challenge and CHIKV pathogenesis in murine models. Additionally, we identified a naturally occurring single residue Lachesin polymorphism that is found in some *Anopheles* (malaria vector) mosquitoes, and fully ablates CHIKV E2–E1 recognition, informing strategies for mosquito-targeted genetic interventions that could prevent mosquito vector infection and virus transmission. These findings define distinct determinants of receptor binding in mosquitoes and humans for arthritogenic alphaviruses, with implications for countermeasure development and outbreak preparedness.

## Full Text Availability

The license terms selected by the author(s) for this preprint version do not permit archiving in PMC. The full text is available from the preprint server.

